# High-mobility group AT-hook 1 promotes cardiac dysfunction in diabetic cardiomyopathy via autophagy inhibition

**DOI:** 10.1038/s41419-020-2316-4

**Published:** 2020-03-02

**Authors:** Qing-Qing Wu, Chen Liu, Zhulan Cai, Qingwen Xie, Tongtong Hu, Mingxia Duan, Haiming Wu, Yuan Yuan, Qizhu Tang

**Affiliations:** 1 Department of Cardiology, Renmin Hospital of Wuhan University, Wuhan, 430060 P. R. China; 2 Cardiovascular Research Institute, Wuhan University, Wuhan, 430060 P. R. China; 3 Hubei Key Laboratory of Cardiology, Wuhan, 430060 P. R. China

**Keywords:** Macroautophagy, Cardiomyopathies

## Abstract

High-mobility group AT-hook1 (HMGA1, formerly HMG-I/Y), an architectural transcription factor, participates in a number of biological processes. However, its effect on cardiac remodeling (refer to cardiac inflammation, apoptosis and dysfunction) in diabetic cardiomyopathy remains largely indistinct. In this study, we found that HMGA1 was upregulated in diabetic mouse hearts and high-glucose-stimulated cardiomyocytes. Overexpression of HMGA1 accelerated high-glucose-induced cardiomyocyte inflammation and apoptosis, while HMGA1 knockdown relieved inflammation and apoptosis in cardiomyocytes in response to high glucose. Overexpression of HMGA1 in mice heart by adeno-associated virus 9 (AAV9) delivery system deteriorated the inflammatory response, increased apoptosis and accelerated cardiac dysfunction in streptozotocin-induced diabetic mouse model. Knockdown of HMGA1 by AAV9-shHMGA1 in vivo ameliorated cardiac remodeling in diabetic mice. Mechanistically, we found that HMGA1 inhibited the formation rather than the degradation of autophagy by regulating P27/CDK2/mTOR signaling. CDK2 knockdown or P27 overexpression blurred HMGA1 overexpression-induced deteriorating effects in vitro. P27 overexpression in mice heart counteracted HMGA1 overexpression-induced increased cardiac remodeling in diabetic mice. The luciferase reporter experiment confirmed that the regulatory effect of HMGA1 on P27 was mediated by miR-222. In addition, a miR-222 antagomir counteracted HMGA1 overexpression-induced deteriorating effects in vitro. Taken together, our data indicate that HMGA1 aggravates diabetic cardiomyopathy by directly regulating miR-222 promoter activity, which inhibits P27/mTOR-induced autophagy.

## Introduction

Epidemiological evidence shows that diabetes mellitus (DM) is strongly associated with heart failure (HF). The Framingham study recorded an incremental incidence of HF in diabetic patients, 2.4-fold in males and 5-fold in females^1^. HF is a more common initial presentation than myocardial infarction in people suffering DM^2^. The development of diabetes also facilitates the evolution of HF in patients with myocardial infarction^3^, hypertension^4^, or atrial fibrillation^5^. Diabetic cardiomyopathy (DCM) is characterized by early diastolic abnormalities and clinical heart failure without dyslipidemia, hypertension, or coronary artery disease^6^. The pathological cardiac remodeling process that drives DCM is complex, which includes systemic metabolic disorders, inflammation, oxidative stress, and apoptosis^7^. These pathologic changes conjointly enhance cardiac tissue interstitial fibrosis, cardiac diastolic dysfunction, and then systolic dysfunction and ultimately clinical HF^6^. Therefore, it is crucial to further research the molecular basis of this remodeling process and elucidate the underlying mechanisms of DCM.

Autophagy is a ubiquitous physiological process that removes harmful aggregated proteins, intracellular pathogens, and damaged organelles through lysosomes^8^. Studies have confirmed the crucial role of autophagy in the development of diabetic cardiomyopathy. In OVE26 mice (develop type 1 diabetic model within 24 h after birth because of beta cell‐specific damage due to a calmodulin transgene regulated by the insulin promoter) cardiac autophagy was inhibited^9^. Suppression of cardiac autophagy was also discovered in STZ-induced diabetic mice^10,11^. In a high-fat diet-induced type 2 DCM mice model (a well-characterized model that results in hyperglycemia, hyperinsulinemia, insulin resistance, defective islet compensation, and impaired glucose tolerance), autophagic flux decreased in myocardial tissue^12^. Studies indicated that restoring cardiac autophagy protected against cardiac remodeling in DCM^9,11^. Thus, regulating autophagy may be a beneficial target for treating DCM.

High-mobility family AT-Hook1 (HMGA1) binds to the AT-rich region of DNA^13^. HMGA1 takes part in various fundamental processes at the molecular and cellular level, such as cell cycle progression^14^, embryologic development^15^, neoplastic transformation^16^, differentiation^17^, apoptotic cellular metabolism, and DNA repair^18^. Research has uncovered that cardiac hypertrophy and myelo-lymphoproliferative disorders were observed in mice with HMGA1 haploinsufficiency^19^. Moreover, HMGA1 was associated with isoprenaline-induced cardiomyocyte hypertrophy^20^. Hopper et al.^21^ found that HMGA1 was upregulated in the pulmonary arterial endothelium in pulmonary arterial hypertension (PAH), and this upregulation promoted endothelial-to-mesenchymal transition and accelerated PAH. These findings suggest that HMGA1 has effects on cardiovascular disease. In this study, we used C57BL/6J mice and neonatal rat cardiomyocytes (NRCMs) since adult mice and rat cardiomyocytes are hard to assess. This study aims to explore the functional role of HMGA1 in streptozotocin-induced diabetic cardiomyopathy and the underlying mechanism.

## Methods

### Ethics approval and consent to participate

All of the animal care and experimental procedures conformed to the Animals (Scientific Procedures) Act 1986, of the UK Parliament, Directive 2010/63/EU of the European Parliament, the Guidelines for the Care and Use of Laboratory Animals published by the United States National Institutes of Health (NIH Publication, revised 2011) and the Guidelines for the Care and Use of Laboratory Animals of the Chinese Animal Welfare Committee; all procedures were approved by the Animal Use Committees of our hospital and our institute. The animal studies also followed the ARRIVE guidelines.

### Animals and animal model

C57/BL6J male mice (8–10 weeks) were purchased from the Chinese Academy of Medical Sciences (Beijing). Less than six mice were housed together in an independently ventilated cage (the area of one cage was 542 cm^2^). The animals had free access to food and water during the study and were housed under a controlled temperature (20–25 °C), humidity (50 ± 5%) and specific pathogen-free environment with a 12 h light/dark cycle. Then, the mice were grouped according to a random number table to receive either streptozotocin (STZ) or control vehicle injection. The mice were subjected to intraperitoneal STZ injection to induce diabetes^10^. Fasting blood glucose (FBG) ≥ 16.6 mmol/l was used to indicate diabetes. The mice were given retro-orbital venous plexus injections of either adeno-associated virus (AAV9)-HMGA1 or AAV9-shHMGA1 to overexpress or knockdown HMGA1 at 10 weeks after STZ injection (*n* = 15 per group). To determine the expression level of HMGA1 in different cell types in heart tissue after AAV9-HMGA1 or AAV9-shHMGA1 injection, mice were directly subjected to retro-orbital venous plexus injections of either AAV9-HMGA1 or AAV9-shHMGA1 for 6 weeks (*n* = 12 per group). Then hearts (*n* = 6) were subjected to Langendorff method to isolate adult mouse cardiomyocytes; other six hearts were subjected to fibroblast isolation. Doppler analysis and invasive hemodynamic measurements were carried out 16 weeks after the STZ injection. The animals were sacrificed with an overdose of sodium pentobarbital, and the hearts were collected for further studies. For mechanism studies, the mice were subjected to retro-orbital venous plexus injection of AAV9-P27 to overexpress P27 at 10 weeks after STZ injection (*n* = 15 per group). After 6 weeks, the mice were subjected to Doppler analysis and invasive hemodynamic measurements; then, the mouse hearts were collected for further study. A random number table method was used for grouping. Animal processing and data analysis were performed blindly.

### Adeno-associated virus vector

Recombinant AAV9s expressing human HMGA1 (AAV9-HMGA1), AAV9-shHMGA1, and AAV9-P27 were constructed by Vigene Bioscience Company (Jinan, China). AAV9-shHMGA1 was constructed with mouse HMGA1 siRNA from Santa Cruz (sc-44334). Briefly, plasmids in which the cytomegalovirus (CMV) promoter drives the expression of human HMGA1, P27 or shHMGA1 were used to generate AAV9-HMGA1, AAV9-P27 or AAV9-shHMGA1 viral particles. AAV9-based vector genomes were cross-packaged into AAV9 capsids via triple transfection of HEK 293T cells, followed by iodixanol gradient centrifugation and FPLC purification. Titers of the AAV vectors (viral genomes/ml) were determined by quantitative real-time PCR. AAV9-LacZ was used as control, which was packed with empty AAV9 virus. A total of 60–80 μl AAV9-HMGA1/AAV9-shHMGA1/AAV9-P27 or AAV9-NC/AAV9-shRNA (5.0–6.5 × 10^13^ VG/ml) was injected into the retro-orbital venous plexus of the mice at 10 weeks after STZ injection as described in a previous study^22^.

### Echocardiographic and hemodynamic data

Echocardiographic and hemodynamic measurements were performed according to our previous studies^23,24^. A MyLab 30CV ultrasound system (Biosound Esaote, Genoa, Italy) and a 10-MHz linear array ultrasound transducer were used in our study for echocardiography measurements. A microtip catheter transducer (SPR-839; Millar Instruments, Houston, TX, United States) was used in our study for cardiac catheterization.

### Histological analysis

All staining was performed as described in our previous study^23,24^. HMGA1, actin, CD45, CD68, and TNF-α antibodies were used for immunohistochemical staining (all from Abcam).

### Cell culture and treatment

Neonatal rat cardiomyocytes were isolated and cultured as described in our previous study^23^. Cells were identified with α-actin staining. NRCMs were infected with Ad-HMGA1 (MOI = 30) for 4 h to overexpress HMGA1. NRCMs were treated with HMGA1 siRNA (Ribo Life Science, Suzhou, China) for 8 h to knock down HMGA1. NRCMs were treated with 33 mM glucose (high glucose); NRCMs were treated with 5.5 mM glucose and 27.5 mM mannitol as a control group. Cardiomyocytes were treated with bafilomycin A1 (100 nM, Sigma) to explore the effect of HMGA1 on autophagy. Cells were transfected with Atg5 siRNA to knockdown Atg5, transfected with Ad-Atg5 to overexpress Atg5 as our previous study described^10^. NRCMs were treated with cyclin-dependent kinase 2 (CDK2) siRNA to knock down CDK2 (Ribo Life Science). NRCMs were transfected with Ad-P27 to overexpress P27. To antagonize miR-222, a miR-222 antagomir (Thermo Fisher Scientific, Waltham, MA, USA) was used. Cell viability was detected by MTT assay. All the in vitro experiments were performed independently for three times.

### Adult mouse cardiomyocyte and fibroblast isolation and culture

The Langendorff method was used to isolate adult mouse cardiomyocytes 6 weeks after the mice received AAV9-HMGA1 or AAV9-shHMGA1 injection. According to a previous study^25^, each mouse was injected intraperitoneally with 100 U of heparin. After 20 min, the heart was rapidly removed, cannulated through the aorta, and mounted on a modified Langendorff perfusion system. The heart was first perfused at a constant flow of 3 ml/min for 3 min with the appropriate buffer, which was then switched to a circulating enzyme digestion solution at 37 °C for 15–20 min until the heart became pale and flaccid. The heart was removed from the perfusion apparatus and dissected, and the atria and large vessels were discarded. The ventricular tissue was disaggregated with forceps and gentle pipetting. The cell suspension was filtered, and the isolated cells were resuspended in 12 ml of fresh stopping buffer. CaCl_2_ from a 100 mM stock solution was slowly added (in four steps over 20 min) to a final concentration of 1 mM. The isolated cardiomyocytes were plated on laminin-coated 35-mm culture dishes. After 2 h of cell adhesion, the plating medium was replaced with culture medium [Minimum Essential Medium (MEM) containing 20 mM hydroxyethyl piperazineethanesulfonic acid (HEPES), 4 mM NaHCO_3_, 0.1 mg/ml bovine serum albumin, 100 U/ml penicillin, 100 μg/ml streptomycin, 2 mM l-glutamine, 1× insulin-transferrin-selenium supplement (Sigma), and 10 mM 2,3-butanedione monoxime (BDM)]. The cells were cultured at 37 °C for 24 h and then collected for western blotting.

#### Adult mouse cardiac fibroblast isolation and culture

Briefly, mice were sacrificed, and their hearts were collected. The hearts were cut into 1-mm^3^ tissue pieces, and 0.125% trypsin and collagenase were used to digest the left ventricles for 15 min at 34 °C for a total of five times. The digestion fluid was collected and centrifuged. The cells were resuspended, filtered and then seeded onto 100-mm plates for 90 min. After removing the cardiomyocytes, the cardiac fibroblasts were cultured in Dulbecco’s Modified Eagle Medium (DMEM)/F12 containing 10% Fetal Bovine Serum (FBS) at 37 °C in a humidified incubator with 5% CO_2_. The cells were cultured at 37 °C for 24 h and then collected for western blotting.

### Real-time PCR and western blotting

As previously described by Xiao, real-time PCR and western blotting were performed^23,24^. The primers used are listed in Table S1, and the antibodies used are listed in Table S2.

### Autophagic flux analysis

NRCMs were transfected with mRFP-GFP-LC3 adenovirus (Ad-tf-LC3, MOI = 50) to detect autophagic flux. The red puncta and green puncta in each group were observed with a fluorescence microscope.

### Luciferase reporter assay

The synthesized promoter regions (−1000 bp and −500 bp) of the miR-222 gene were subcloned into the luciferase reporter vector (Promega, USA). Luciferase reporter constructs were packed with an adenoviral system and then cotransfected into NRCMs with a control plasmid, followed by the indicated stimulation: Ad-HMGA1 transfection or siHMGA1 for 48 h. Then, the cells were harvested and lysed, and the luciferase activity was determined with the Dual-Luciferase Reporter Assay Kit (Promega, USA) according to the manufacturer’s instructions. The PGL3 basic vector was used as a negative control.

### Statistical analyses

All data are presented as the mean ± SD. SPSS23.0 was used for analyses. Differences between multiple groups were analyzed by one-way ANOVA. Differences between two groups were analyzed by unpaired Student’s *t* test^26^. Statistical significance was defined as a *P* value less than 0.05. In accordance with our previously published article, the method of power analysis for body weight with an *α* error of 5% and a power of 80% was used to determine the in vivo group sizes^27^. No samples were excluded from the analyses.

## Results

### HMGA1 expression pattern in DCM hearts

**Fig. 1:**
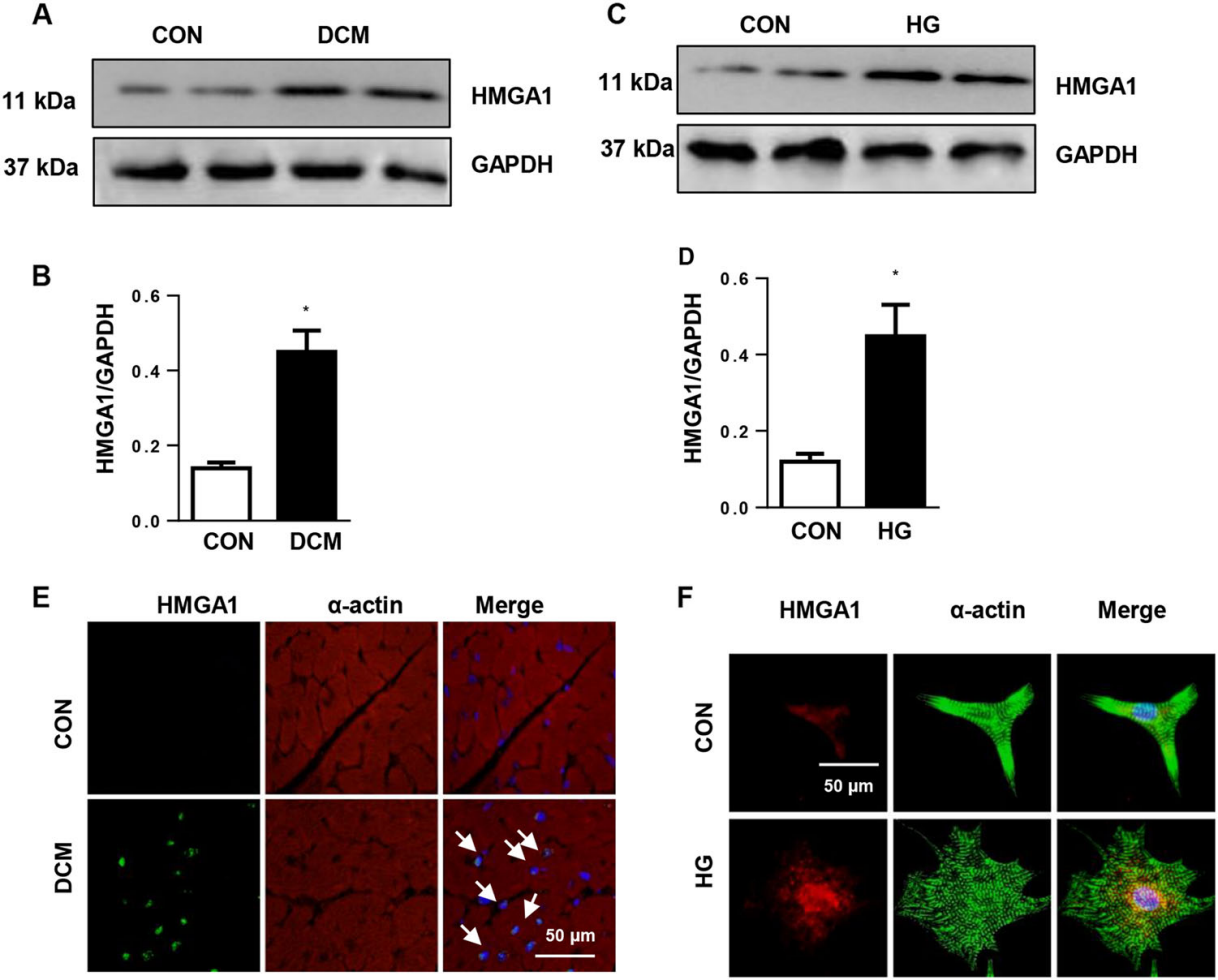
Expression levels of HMGA1 in DCM hearts. **a**, **b** Protein expression of HMGA1 in DCM mouse hearts (*n* = 6 samples per group, **a** and **b** from the same sample. *P < 0.05 vs. the control (CON) group). **c**, **d** Western blot image and quantification of HMGA1 in high-glucose (HG)-stimulated neonatal rat cardiomyocytes (NRCMs) (*n* = 6, *P < 0.05 vs. the CON group). **e** HMGA1 and α-actin staining in diabetic cardiomyopathy (DCM) mouse hearts (*n* = 5 samples per group). **f** HMGA1 and α-actin staining in HG-stimulated NRCMs (*n* = 5). All the in vitro experiments were performed independently for three times.

To explore whether the expression level of HMGA1 changes in the pathology of DCM, we first detected HMGA1 expression in DCM mouse hearts and high-glucose (HG)-stimulated cardiomyocytes. HMGA1 was upregulated in DCM mouse hearts (Fig. 1a, b) as well as HG-stimulated cardiomyocytes (Fig. 1c, d). Immunofluorescence staining result showed that HMGA1 was located in the nuclei and increased in DCM heart tissue (Fig. 1e) and insult cardiomyocytes (Fig. 1f). These alterations indicate that HMGA1 may be associated with the remodeling process in DCM hearts.

### HMGA1 promotes inflammation and apoptosis in cardiomyocytes

**Fig. 2:**
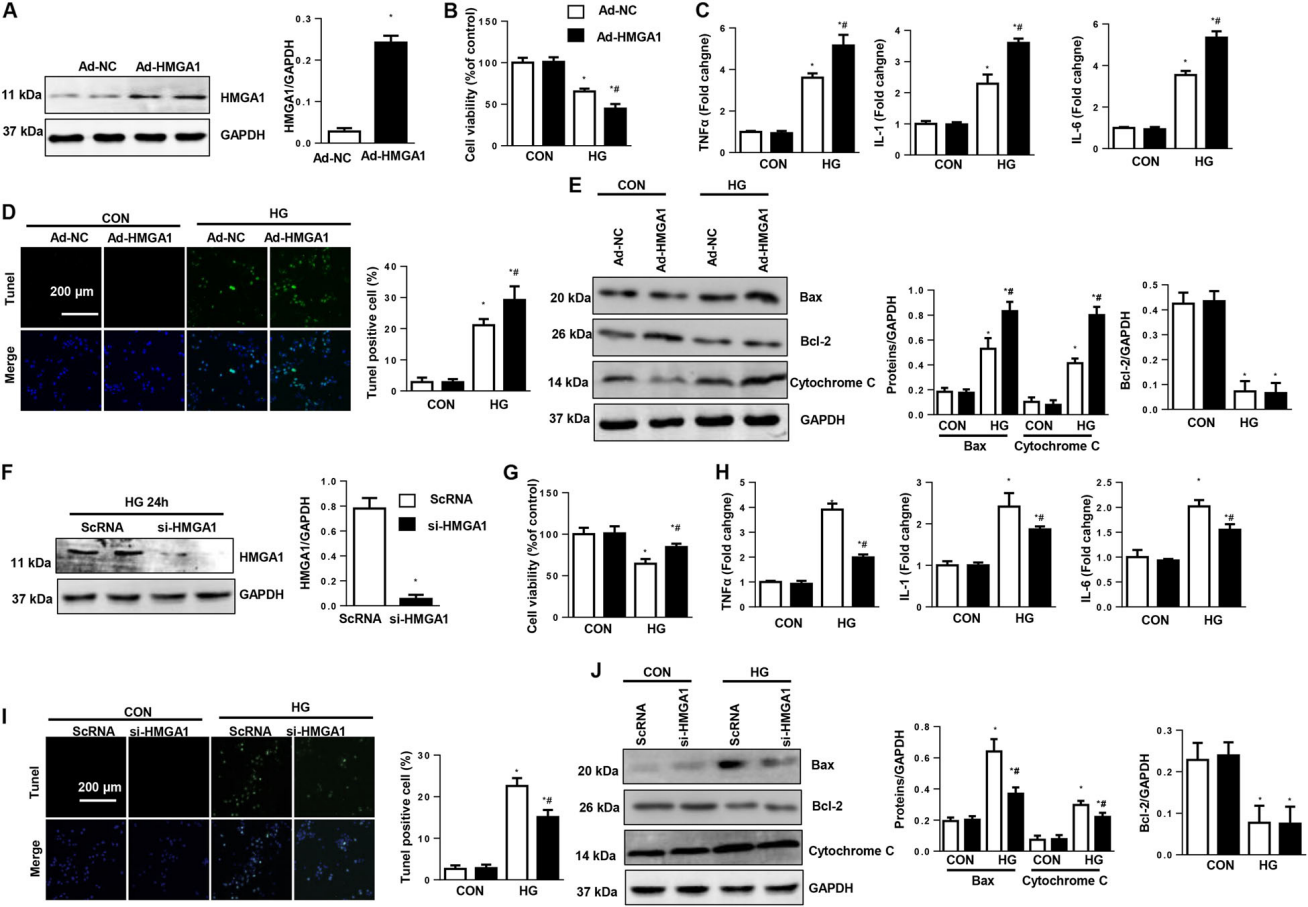
HMGA1 promotes high-glucose-induced inflammation and apoptosis in vitro. **a** Western blot image and quantification of HMGA1 in NRCMs transfected with Ad-HMGA1 (*n* = 6, *P < 0.05 vs. the Ad-NC group). **b**–**e** NRCMs were transfected with Ad-HMGA1 and then stimulated with HG for 48 h. **b** Cell viability detected by MTT assay in NRCMs in the indicated group (*n* = 6). **c** mRNA expression of proinflammatory markers in NRCMs (*n* = 6). **d** TUNEL staining and quantification results in NRCMs (*n* = 5). **e** Western blot image and quantification of Bax, Bcl-2, and cytochrome C (*n* = 6). *P < 0.05 vs. the Ad-NC-CON group; ^#^*P* < 0.05 vs. the Ad-NC-HG group. **f** Western blot image and quantification of HMGA1 in NRCMs transfected with HMGA1 siRNA (*n* = 6, *P < 0.05 vs. the ScRNA group). **g**–**j** NRCMs were transfected with HMGA1 siRNA and then stimulated with HG for 48 h. **g** Cell viability detected by MTT assay in NRCMs (*n* = 6). **h** mRNA levels of proinflammatory markers in NRCMs (*n* = 6). **i** TUNEL staining and quantification results in NRCMs (*n* = 5). **j** Protein expression of Bax, Bcl-2, and cytochrome C (*n* = 6). *P < 0.05 vs. the ScRNA-CON group; ^#^*P* < 0.05 vs. the ScRNA-HG group. All the in vitro experiments were performed independently for three times.

To explore the function of HMGA1 in the development of DCM, NRCMs were infected with Ad-HMGA1 (Fig. 2a). HG stimulation decreased cell viability and increased inflammatory cytokine expression and cell apoptosis as evidenced by increased TUNEL-positive cell numbers, Bax-to-Bcl-2 ratio imbalance and augmented cytochrome C release. HMGA1 overexpression further reduced cell viability and promoted HG-induced cell inflammation and apoptosis, but the expression of Bcl-2 was unchanged (Fig. 2b–e). We then knocked down HMGA1 with HMGA1 siRNA (Fig. 2f). The decreased cell viability was increased by HMGA knockdown (Fig. 2g). The inflammatory response and apoptosis levels were also lower in the HMGA1-silenced group than in the ScRNA group (Fig. 2h–j). The expression of Bcl-2 was still unchanged in HMGA1 knockdown cells. All these data suggest that HMGA1 accelerates HG-induced cell damage.

### HMGA1 overexpression accelerates cardiac remodeling in vivo

**Fig. 3:**
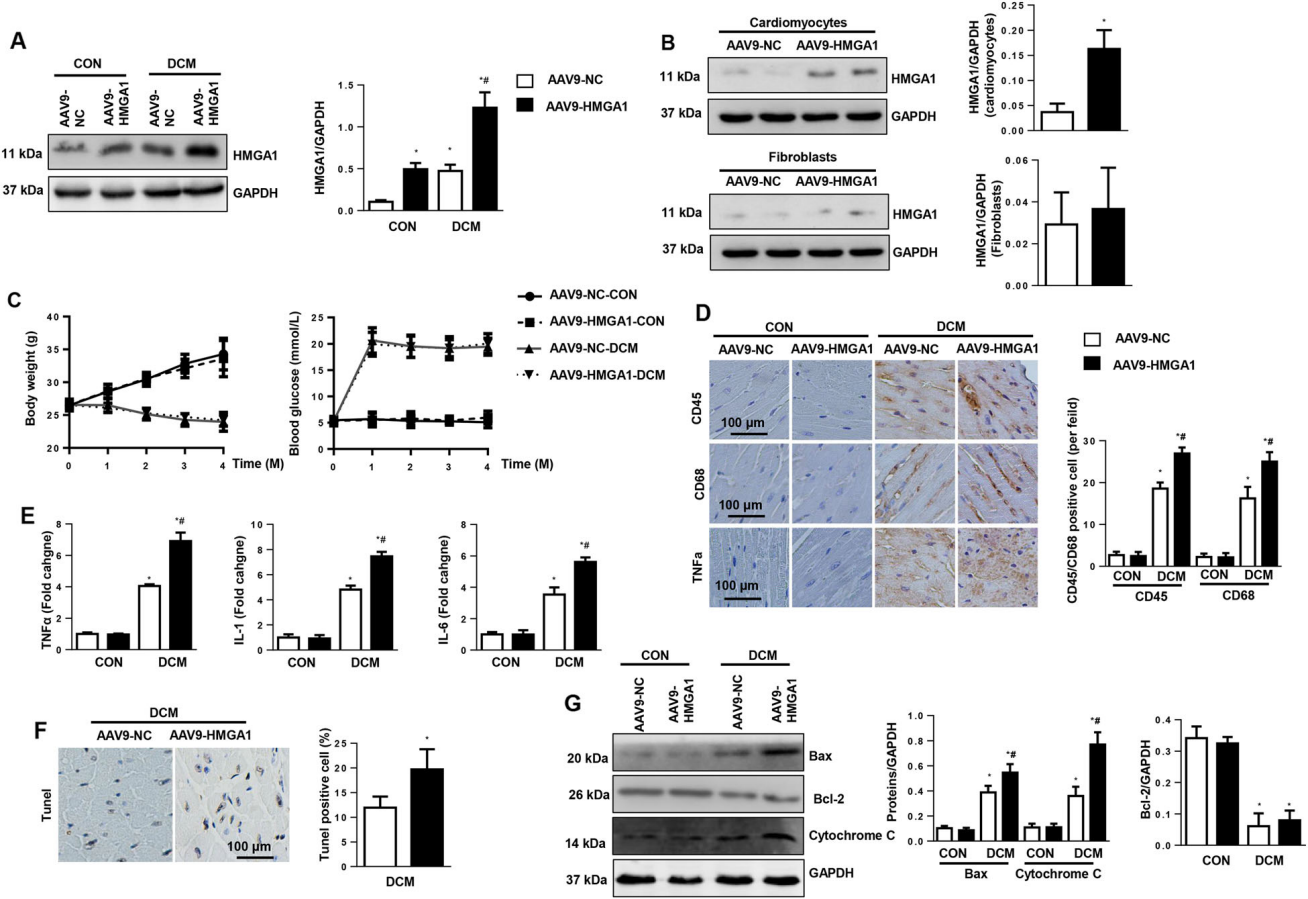
HMGA1 overexpression accelerates cardiac remodeling in DCM mouse hearts. **a**, **c**–**g** Mice received an AAV9-HMGA1 injection at 10 weeks after the final STZ injection (*n* = 12 per group). **a** Protein levels of HMGA1 in mouse hearts 6 weeks after AAV9-HMGA1 injection and with or without STZ injection (*n* = 6 mice hearts). **b** Mice received an AAV9-HMGA1 injection for 6 weeks. The expression of HMGA1 in cardiomyocytes (*n* = 6) and fibroblasts (*n* = 6) isolated from heart tissues. **c** Body weights and blood glucose levels in DCM mice 0, 1, 2, 3, and 4 months after the final STZ injection (*n* = 12 sample). **d** Immunohistochemical staining and quantification results for CD45, CD68, and TNFα in DCM mouse hearts (*n* = 5). **e** mRNA levels of proinflammatory markers in DCM mouse hearts (*n* = 6). **f** TUNEL staining and quantification results in DCM mouse hearts (*n* = 5). **g** Protein levels of Bax, Bcl-2, and cytochrome C in DCM mouse hearts (*n* = 6). *P < 0.05 vs. the AAV9-NC-CON group; ^#^*P* < 0.05 vs. the AAV9-NC-DCM group.

The functional role of HMGA1 in the DCM mouse model was determined. Mice were subjected to STZ injection to establish a diabetes model and then subjected to AAV9-HMGA1 injection to overexpress HMGA1 (Fig. 3a). To determine the expression level of HMGA1 in different cell types in heart tissue after AAV9-HMGA1 injection, cardiomyocytes and fibroblasts were isolated from mouse hearts 6 weeks after AAV9-HMGA1 injection. As a result, HMGA1 protein levels were increased in isolated cardiomyocytes. Increased HMGA1 was also observed in fibroblasts, but this change was not significantly different (Fig. 3b). During the 4 months of the pathological process, the body weights in the DCM group decreased, and the blood glucose levels increased compared with those in the control group. HMGA1 overexpression did not affect mouse body weights and blood glucose levels (Fig. 3c). We then detected the inflammation and apoptosis levels in DCM mouse hearts. As a result, an increased inflammatory response was observed in mouse hearts 16 weeks after STZ injection, as evidenced by increased CD45-labeled leukocyte and CD68-labeled macrophage infiltration, as well as increased proinflammatory cytokine release (Fig. 3d, e). These inflammatory responses were also greater in HMGA1-overexpressing mice than in mice receiving AAV9-NC injection (Fig. 3d, e). Moreover, TUNEL-positive cardiomyocytes were increased in DCM mouse hearts, while this effect was further increased in mice receiving AAV9-HMGA1 injection. Consistently, the imbalance in the Bax/Bcl-2 ratio and increased release of cytochrome C were augmented in the hearts from HMGA1-overexpressing DCM mice (Fig. 3f, g).

We also evaluated cardiac function with echocardiography and pressure-volume loop. As a result, the left ventricular (LV) ejection fraction (LVEF), LV fractional shortening (LVFS), dp/dtmax, and dp/dtmin were decreased, and the Tau value was higher in the DCM mouse hearts than in the control mouse hearts. HMGA1 overexpression further reduced LVEF, LVFS, dp/dtmax, and dp/dtmin and increased the Tau value. The heart rate remained the same in all four groups (Table S3). HMGA1 overexpression did not change the cardiac function or geometry under physiological condition.

### HMGA1 knockdown inhibits cardiac remodeling in DCM mouse hearts

To explore whether HMGA1 silencing would alleviate the cardiac remodeling process in DCM, mice were injected with AAV9-shHMGA1 to knockdown HMGA1 (Fig. S1a). To determine the expression level of HMGA1 in different cell types in heart tissue after AAV9-shHMGA1 injection, cardiomyocytes and fibroblasts were isolated from mouse hearts 6 weeks after AAV9-shHMGA1 injection. As a result, HMGA1 protein levels were reduced in isolated cardiomyocytes (Fig. S1b). The expression level of HMGA1 in fibroblasts was not significantly different between AAV9-shHMGA1 and AAV9-NC group (Fig. S1b). The body weight and blood glucose levels were not significantly different between the AAV9-shHMGA1-DCM group and the AAV9-shRNA-DCM group (Fig. S1c). Expectedly, the inflammatory response and cardiomyocyte apoptosis were reduced upon HMGA1 knockdown when compared with those in the AAV9-shRNA-DCM group (Fig. S1d–g). Moreover, HMGA1 silencing reduced cardiac dysfunction as evidenced by increased LVEF, FS, dp/dtmax, and dp/dtmin and a decreased Tau value in the AAV9-shHMGA1 group when compared with those in the AAV9-shRNA-DCM group (Table S2). The heart rate also remained the same in all four groups (Table S4). Furthermore, HMGA1 knockdown did not change the cardiac function or geometry under physiological condition.

### HMGA1 regulates autophagy in NRCMs

**Fig. 4:**
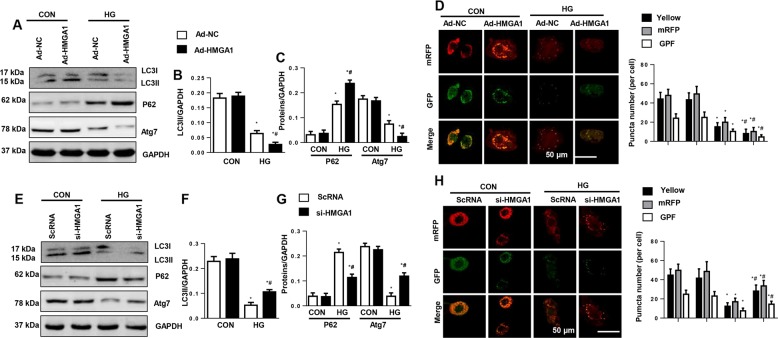
HMGA1 regulates autophagy in NRCMs. **a**–**c** Protein levels of LC3, P62, and Atg7 in NRCMs transfected with Ad-HMGA1 (*n* = 6). **d** NRCMs were transfected with mRFP-GFP-LC3-adenovirus and Ad-HMGA1 and then stimulated with HG for 48 h. mRFP and GFP fluorescence and quantitated yellow puncta number per cell (*n* = 5). *P < 0.05 vs. the Ad-NC-CON group; ^#^*P* < 0.05 vs. the Ad-NC-HG group. **e**–**g** Western blot image and quantification of LC3, P62, and Atg7 in NRCMs transfected with HMGA1 siRNA (*n* = 6). **h** NRCMs were transfected with mRFP-GFP-LC3-adenovirus and HMGA1 siRNA and then stimulated with HG for 48 h. mRFP and GFP fluorescence and quantitated red, green, yellow puncta number per cell (*n* = 5). *P < 0.05 vs. the ScRNA-CON group; ^#^*P* < 0.05 vs. the ScRNA-HG group. All the in vitro experiments were performed independently for three times.

As autophagy impairment accelerates the pathological progress of DCM^10,28^, we detected the association of HMGA1 and autophagy. HG stimulation reduced the autophagy levels as assessed by a decreased LC3II to LC3I ratio and augmented P62 accumulation (Fig. 4a–c). To detect autophagic flow, NRCMs were infected with Ad-mRFP-GFP-LC3. Consistently, autophagic flux was reduced in HG-stimulated NRCMs, as evidenced by the decreased number of red and yellow puncta number (Fig. 4d). Unexpectedly, HMGA1 overexpression further decreased autophagy levels and autophagic flux (Fig. 4a–d). The autophagy level was also detected in HMGA1-silenced NRCMs. Conversely, HMGA1 knockdown increased autophagy levels and increased autophagic flux (Fig. 4e–h).

### Autophagy mediates the effects of HMGA1 in cardiomyocytes

**Fig. 5:**
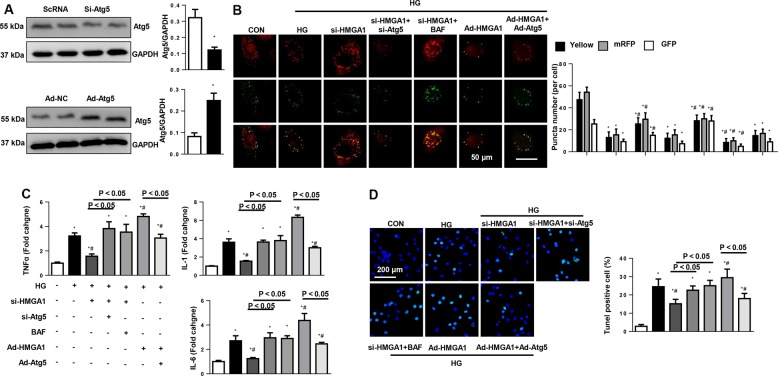
Autophagy mediates the functional role of HMGA1 in cardiomyocytes. NRCMs were transfected with HMGA1 siRNA, Atg5 siRNA, treated with bafilomycin A1 (Baf) for 2 h and then stimulated with HG for 48 h. NRCMs were transfected with Ad-HMGA1, Ad-Atg5, and treated with rapamycin (RAP) for 2 h, and then stimulated with HG for 48 h. **a** The expression level of Atg5 when cells were transfected with Atg5 siRNA or Ad-Atg5 (*n* = 6)**. b** NRCMs were transfected with mRFP-GFP-LC3-adenovirus. mRFP and GFP fluorescence and quantitated red, green, yellow puncta number per cell (*n* = 5). **c** Transcription levels of proinflammatory markers in NRCMs in the indicated group (*n* = 6). **d**, **e** TUNEL staining and quantification results in NRCMs in the indicated groups (*n* = 5). *P < 0.05 vs. the CON group; ^#^*P* < 0.05 vs. the HG group. All the in vitro experiments were performed independently for three times.

Then, to determine whether autophagy regulation mediated the proremodeling effects of HMGA1, NRCMs were transfected with Atg5 siRNA to knockdown Atg5, and transfected with Ad-Atg5 to overexpress Atg5 (Fig. 5a), or treated with autophagy inhibitors bafilomycin A1 (Baf, inhibits the degradation of autophagy lysosomes). HMGA1 knockdown increased the autophagy flux (enhanced red puncta number and the yellow puncta number). When blocking autophagy degradation with bafilomycin A1, autophagy flux was blocked in HMGA1-silenced cells as evidenced with more green puncta number in cells. When inhibiting autophagy with Atg5 siRNA, the autophagy flux was decreased in HMGA1-silenced cells. Atg5 overexpression could save the diminished autophagy flux in HMGA1-overexpressed cells (Fig. 5b). These data indicate that HMGA1 regulates the formation of autophagy rather than the degradation of autophagy. We found that the Atg5 silence and Baf counteracted the protective effects of HMGA1 knockdown (decreased inflammation and apoptosis levels), while the Atg5 overexpression ameliorated the HMGA1 overexpression-induced deterioration phenotype (Fig. 5c, d). These results suggest that by regulating autophagy, HMGA1 accelerates cardiomyocyte injury.

### HMGA1 regulates P27/CDK2/mammalian target of rapamycin (mTOR) signaling

**Fig. 6:**
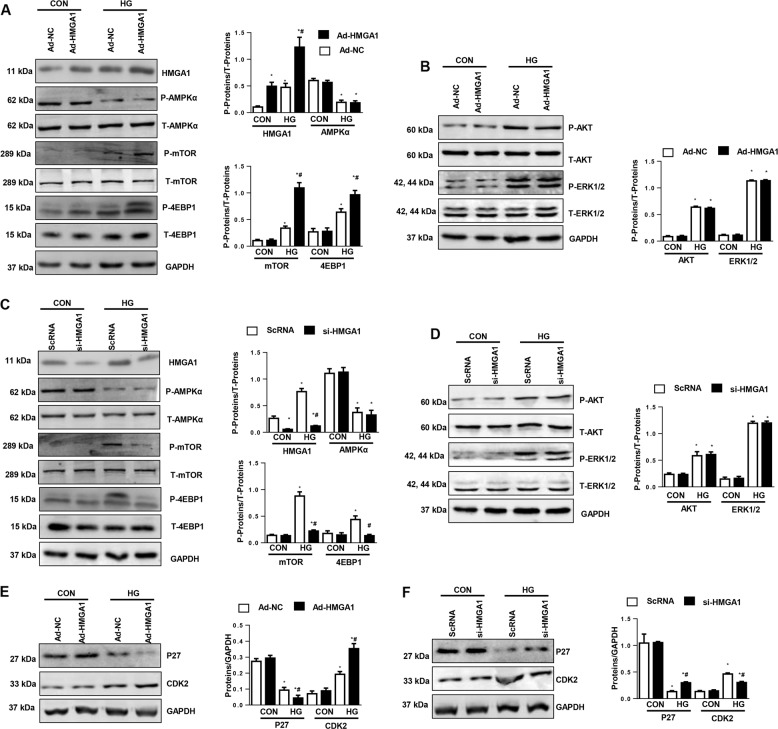
HMGA1 regulates P27/CDK2/mTOR signaling. **a**, **b**, **e** Western blot image and quantification of phosphorylated (P-) and total (T-) AMPKα, mTOR, 4EBP1, AKT, ERK1/2, P27, and CDK2 in NRCMs transfected with Ad-HMGA1 (*n* = 6, *P < 0.05 vs. the Ad-NC-CON group; ^#^*P* < 0.05 vs. the Ad-NC-HG group). **c**, **d**, **f** Western blot image and quantification of phosphorylated and total AMPKα, mTOR, 4EBP1, AKT, ERK1/2, P27, and CDK2 in NRCMs transfected with HMGA1 siRNA (*n* = 6, *P < 0.05 vs. the ScRNA-CON group; ^#^*P* < 0.05 vs. the ScRNA-HG group). All the in vitro experiments were performed independently for three times.

We then investigated the detailed mechanism through which HMGA1 regulates autophagy levels. We found that phosphorylated-mTOR, an autophagy-inhibiting protein, and the downstream protein 4EBP1 were increased in HMGA1-overexpressing NRCMs and reduced in HMGA1-silenced NRCMs (Fig. 6a–d). However, neither HMGA1 overexpression nor HMGA1 knockdown affected the phosphorylation level of adenosine 5′-monophosphate (AMP)-activated protein kinase (AMPK)α, AKT, or ERK1/2, the three upstream proteins of mTOR (Fig. 6a–d). Studies have reported that P27/CDK2 regulates mTOR activation. We then detected P27 signaling. Interestingly, decreased P27 levels were observed in HG-stimulated NRCMs. HMGA1 overexpression decreased P27 levels and increased CDK2 levels, whereas HMGA1 silencing increased P27 levels and decreased CDK2 expression (Fig. 6e, f).

### P27/CDK2 mediates the functional role of HMGA1 in vitro

Cardiomyocytes were treated with CDK2 siRNA to knock down CDK2 (Fig. S2a). CDK2 silencing did not affect the expression level of HMGA1 (Fig. S2a, b) but did abrogate the deteriorating effects of HMGA1 overexpression on cardiomyocytes (Fig. S2c, d). NRCMs were transfected with CDK2 siRNA to overexpress P27 (Fig. S2e). P27 overexpression did not affect the expression level of HMGA1 (Fig. S2e, f) but did counteract the protective effects of HMGA1 silencing on cardiomyocytes (Fig. S2g, h).

### Overexpression of P27 counteracts the effects of HMGA1 in DCM mouse hearts

**Fig. 7:**
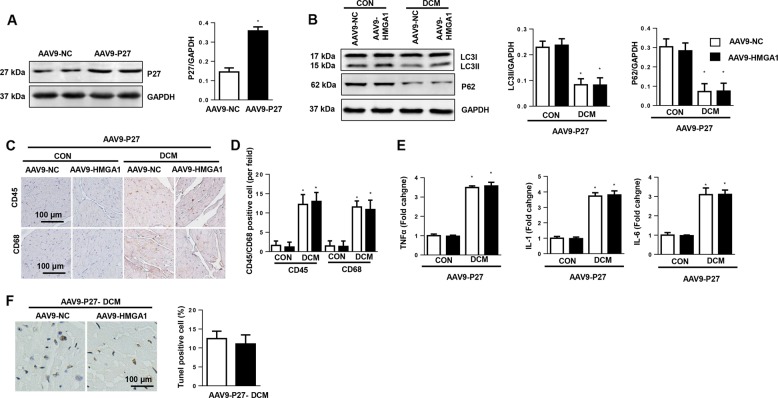
P27 overexpression counteracts the effects of HMGA1 in DCM mouse hearts. Mice received AAV9-HMGA1 and AAV9-P27 injections at 10 weeks after the final STZ injection (*n* = 12 per group). **a** Protein levels of P27 in mouse hearts 6 weeks after AAV9-P27 injection (*n* = 6). **b** Protein levels of LC3 and P62 in DCM mouse hearts (*n* = 6). **c**, **d** Immunohistochemical staining and quantification results for CD45, CD68 in DCM mouse hearts (*n* = 5). **e** Transcription levels of proinflammatory markers in DCM mouse hearts (*n* = 6). **f** TUNEL staining and quantification results in mouse hearts (*n* = 5). *P < 0.05 vs. the AAV9-NC-CON group.

To confirm the causal relationship of P27 in HMGA1-mediated effects in vivo, mice were injected with AAV9-HMGA1 and AAV9-P27 to overexpress HMGA1 and P27 (Fig. 7a). As a result, the effects of HMGA1 on autophagy levels were blocked by P27 overexpression (Fig. 7b). P27 overexpression also counteracted the deteriorating phenotype induced by HMGA1 overexpression (Fig. 7c–f; Table S5).

### HMGA1 regulates P27 via miR-222

**Fig. 8:**
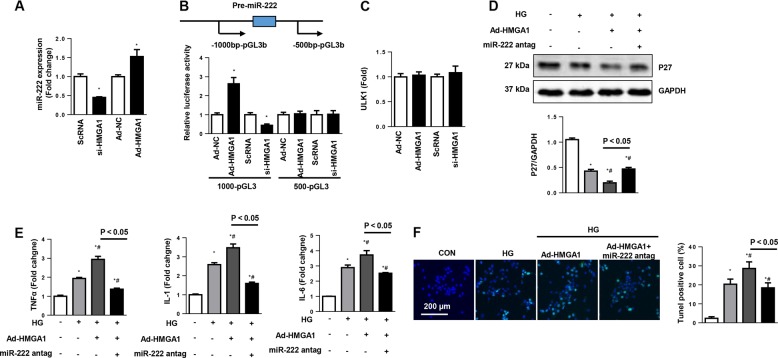
HMGA1 regulates P27 via miR-222. **a** miR-222 expression levels in NRCMs transfected with Ad-HMGA1 or HMGA1 siRNA (*n* = 6, *P < 0.05 vs. the ScRNA/Ad-NC group)**. b** Relative luciferase activity of miR-222 in NRCMs transfected with Ad-HMGA1 or HMGA1 siRNA. **c** ULK1 mRNA levels in NRCMs transfected with Ad-HMGA1 or HMGA1 siRNA (*n* = 6). **d**–**f** NRCMs were transfected with Ad-HMGA1, treated with miR-222 antagomir, and then stimulated with HG for 48 h. **d** Protein expression levels of P27 in the indicated group (*n* = 6). **e** Transcription levels of proinflammatory markers in NRCMs (*n* = 6). **f** TUNEL staining and quantification results in NRCMs (*n* = 5). *P < 0.05 vs. the CON group; ^#^*P* < 0.05 vs. the HG group. All the in vitro experiments were performed independently for three times.

Studies reported that HMGA1 regulated miR-222 in cervical cancer cells^29^. In addition, miR-222 inhibits P27 promoter activity^30^. We then detected the association between HMGA1 and miR-222 in cardiomyocytes. HMGA1 knockdown reduced miR-222 expression, while HMGA1 overexpression increased miR-222 expression levels (Fig. 8a). We then hypothesized that HMGA1 may regulate miR-222 transcription. To explore whether HMGA1 affects the promoter activity of miR-222, 1000-pGL3b and 500-pGL3b reporter plasmids were constructed, packed into adenoviral delivery system and transfected into NRCMs. The results showed that the HMGA1 increased the luciferase activity in NRCMs transfected with the 1000-pGL3b miR-222 binding site plasmid but not in NRCMs transfected with the 500-pGL3b miR-222 binding site plasmid. Moreover, HMGA1 siRNA reduced the luciferase activity in NRCMs transfected with the 1000-pGL3b miR-222 binding site plasmid (Fig. 8b). A previous study reported that HMGA1 influenced the level of ULK1, thus modulating autophagy in HeLa cells^31^. We then detected whether HMGA1 affected ULK1 in cardiomyocytes. As shown in Fig. 8c, neither HMGA1 overexpression nor HMGA1 knockdown affected the transcription level of ULK1. A miR-222 antagomir was used to confirm the direct effect of HMGA1. The protein level of P27 was reduced by HMGA1 overexpression but increased by treatment with the miR-222 antagomir (Fig. 8d). The proinflammatory and proapoptotic effects of HMGA1 were also blocked by the miR-222 antagomir (Fig. 8e, f).

## Discussion

The HMGA1 protein is a structural factor that binds to DNA regions rich in adenine-thymine (A-T), which have no intrinsic transcriptional activity^16^. HMGA1 drives gene transcription by promoting the assembly and stability of higher transcriptional complexes^18^. Previous studies demonstrated that HMGA1 deficiency induces cardiac hypertrophy^19,20^. In our study, we found that HMGA1 was highly upregulated in DCM mouse hearts and HG-stimulated cardiomyocytes. HMGA1 overexpression caused deteriorating cardiac inflammation, apoptosis and dysfunction in DCM mice, while HMGA1 knockdown retarded cardiac inflammation, apoptosis and dysfunction in DCM mice. These functional effects of HMGA1 on DCM were dependent on the regulation of miR-222 promoter activity, which inhibited the P27/mTOR-mediated autophagy.

The process in which long-lived proteins and organelles are delivered to lysosomes for degradation is called autophagy, a cellular catabolic pathway^8^, which at its basal level, plays a role in housekeeping and maintaining cellular homeostasis^32^. Recent studies prove that a basal level of autophagy is needed to maintain normal cardiac function and morphology^33,34^. Autophagy disruption causes cardiac dysfunction in aged mice^33^ and aggravates cardiac remodeling and heart failure caused by stress overload^34^. However, imbalanced autophagy seems to be harmful to the heart, as evidenced by the damage caused by diphtheria toxin and the anticancer drug doxorubicin^35,36^. Thus, depending on the specific circumstances, autophagy may be protective or harmful. In type 1 DCM hearts, including the OVE26 and STZ-induced models, autophagy is decreased rather than increased^9,10^. However, the results are inconsistent in type 2 diabetes. In high-fat diet-induced obesity and metabolic syndrome, autophagy is decreased^12^. In fructose-induced insulin resistance and hyperglycemia, autophagy is increased^37^. Increased autophagy was reported to inhibit cardiac remodeling in STZ-induced DCM^10^. A previous study reported that HMGA1 negatively regulated autophagy in skin cancer cells^31^. In this study, we found that autophagy flux was decreased in type 1 DCM hearts and in cardiomyocytes exposed to high glucose. HMGA1 decreased autophagy but did not affect the degradation of autophagy. Increasing autophagy by rapamycin could block HMGA1-induced cardiac injury effects. While decreasing autophagy by 3-MA and bafilomycin A1 counteracted the antiremodeling effects of HMGA1 silencing. Our data indicate that HMGA1 deteriorated cardiac remodeling in DCM hearts by inhibiting autophagy.

Autophagy could be regulated by several factors such as AMPK and mTOR^32^, which commence with the activation of the ULK1 complex, which mediates the phosphorylation of Atg13 and FIP200 to induce autophagy^32^. Under nutrient-rich conditions, mTOR inhibits ULK1, thereby inhibiting autophagy. However, under stimulating conditions, ADP:AMP accumulation activates AMPK, negatively regulating mTOR^28^. Inactivated mTORC1 promotes the formation of proautophagic complexes, leading to the formation of autophagosomes^32^. In our study, we found that HMGA1 inhibited autophagy formation but not degradation. In addition, HMGA1 increased mTOR activation, thus causing autophagy inhibition. We detected three molecules upstream of mTOR: AKT^38^, AMPKα^27^, and ERK1/2^39^. We found that all of these upstream proteins were not affected by HMGA1. By downregulating Bcl-2 (an autophagy inhibitor) through the Rb/E2F1 axis, P27 is considered to be an autophagy protein^40^. Recently, Su et al.^41^ found that mTOR activity was tightly regulated by P27/CDK2 in cardiomyocytes. We found that both P27 and Bcl-2 were decreased in both DCM mouse hearts and HG-stimulated cardiomyocytes. Contrary to expectations, Bcl-2 was not affected by HMGA1. But P27 was reduced in HMGA1-overexpressing cardiomyocytes and augmented in HMGA1-silenced cardiomyocytes. As a CDK inhibitor, P27 interacts with CDK2 to inhibit the cell cycle^42^. Consistent with P27 levels, CDK2 expression was increased by HG stimulation and further increased in HMGA1-overexpressing cardiomyocytes. We used CDK2 siRNA to knockdown CDK2 and Ad-P27 to overexpress P27 in cardiomyocytes and found that the cardiac injury phenotype in cardiomyocytes overexpressing HMGA1 was blocked by CDK2 knockdown. While the cardiac protection phenotype in HMGA1-silenced cardiomyocytes and heart tissue was counteracted by P27 overexpression. Conte A reported that HMGA1 could influence the level of ULK1, thus modulating autophagy in HeLa cells^31^. In our study, we focused on the effect of HMGA1 on cardiomyocytes and did not observe an effect of HMGA1 on ULK1. These data indicate that by negatively regulating P27/CDK2/mTOR in cardiomyocytes, HMGA1 inhibits autophagy, thus inhibiting cardiac remodeling in DCM hearts.

By inhibiting target mRNA translation or promoting mRNA degradation, miRNAs regulate many physiological and pathological processes^43^. Numerous studies have confirmed that microRNAs participate in the pathology of many cardiovascular diseases, such as cardiac remodeling, heart failure, myocardial damage, and DCM^43–45^. microRNAs could become potential therapeutic targets or prognostic indicators^43–45^. Studies reported that miR-222 was negatively regulated in vascular smooth muscle cells and many cancer cell lines^30,46^. Zhang et al.^29^ found that miR-222 was positively regulated by the HMGA1 protein in lung cancer cells. In our study, we found that miR-222 was reduced by HMGA1 knockdown but increased by HMGA1 overexpression. Luciferase reporter results showed that HMGA1 directly regulated the promoter region of miR-222. A miR-222 antagomir could enhance the reduced P27 expression in HMGA1-overexpressing cardiomyocytes and even counteracted the cardiac injury effects of HMGA1 overexpression. These findings indicate that by positively regulating miR-222 promoter activity, HMGA1 inhibits P27/CDK2/mTOR-induced autophagy. In our study, we found that under normal physical conditions, HMGA1 overexpression by itself did not have any effects on cardiomyocytes, although HMGA1 affected miR-222 expression in nonstimulation conditions. These findings indicate that only under pathological conditions, HMGA1 overexpression accelerates the pathological development of DCM.

## Conclusion

In summary, we found that HMGA1 participates in the cardiac remodeling process during DCM via miR-222-dependent regulation of P27/CDK2/mTOR-mediated autophagy. Thus, new therapeutic modalities targeting HMGA1 may have potential for treating DCM.

## Supplementary information

Supplementary Materials

Supplementary Materials

Supplementary Materials-1

Supplementary Material-2

## Data Availability

The datasets used and/or analyzed in the current study are available from the corresponding authors upon reasonable request.
