# Supplementary figures and images for: High-mobility group AT-hook 1 promotes cardiac dysfunction in diabetic cardiomyopathy via autophagy inhibition
Source: Cell Death Dis. 2020 Mar 2;11(3):160. doi: 10.1038/s41419-020-2316-4 (PMC7052237; doi:10.1038/s41419-020-2316-4)

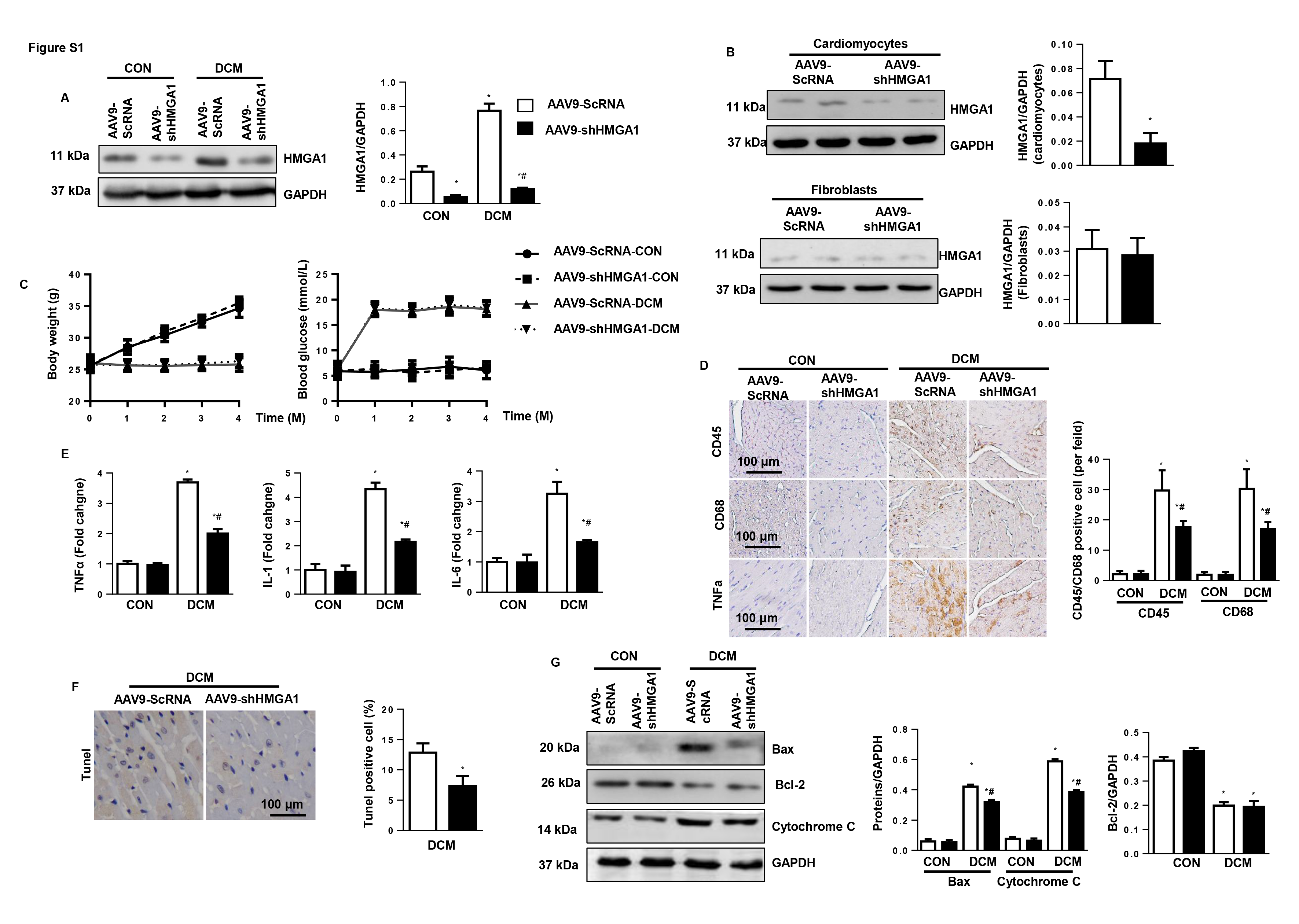

Supplement: Supplementary file 1 — Supplementary Materials [file 41419_2020_2316_MOESM1_ESM.tif]

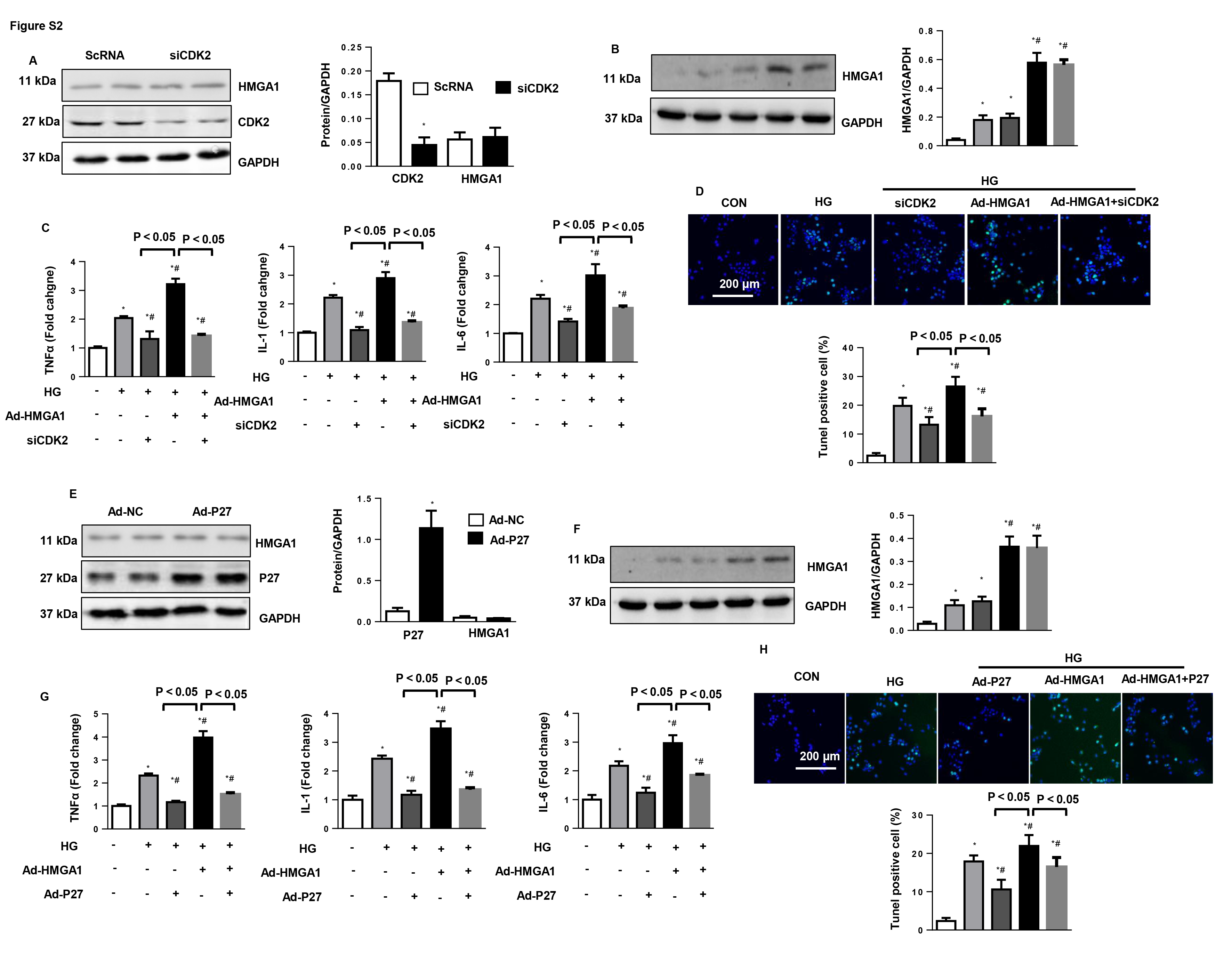

Supplement: Supplementary file 2 — Supplementary Materials [file 41419_2020_2316_MOESM2_ESM.tif]
