# Supplementary material for: High-mobility group AT-hook 1 promotes cardiac dysfunction in diabetic cardiomyopathy via autophagy inhibition
Source: Cell Death Dis. 2020 Mar 2;11(3):160. doi: 10.1038/s41419-020-2316-4 (PMC7052237; doi:10.1038/s41419-020-2316-4)
Supplement: Supplementary file 3 — Supplementary Materials-1 [file 41419_2020_2316_MOESM3_ESM.docx]

**Supplementary materials**

**Figure legends**

**Figure S1. HMGA1 knockdown inhibits cardiac remodeling in DCM mouse hearts**

**A, C-G.**Mice received an AAV9-shHMGA1 injection at 10 weeks after the final STZ injection (n=12 per group). **A.** Protein levels of HMGA1 in mouse hearts 6 weeks after AAV9-shHMGA1 injection and with or without STZ injection (n=6 mice hearts). **B.** Mice were received an AAV9-shHMGA1 injection for 6 weeks. The expression of HMGA1 in cardiomyocytes (n=6) and fibroblasts (n=6) isolated from heart tissues. **C**. Body weights and blood glucose levels in DCM mice 0, 1, 2, 3, and 4 months after the final STZ injection (n=12). **D.** Immunohistochemical staining and quantification results for CD45, CD68, and TNFα in DCM mouse hearts (n=5). **E.** mRNA levels of pro-inflammatory markers in DCM mouse hearts (n=6). **F.** TUNEL staining and quantification results in DCM mouse hearts (n=5). **G.** Protein levels of Bax, Bcl-2, and cytochrome C in DCM mouse hearts (n=6). *P<0.05 *vs.* the AAV9-ScRNA-CON group; #P<0.05 *vs.* the AAV9-ScRNA-DCM group.

**Figure S2. P27/CDK2 mediates the functional role of HMGA1 in cardiomyocytes**

**A-D.** NRCMs were transfected with CDK2 siRNA and Ad-HMGA1 and then stimulated with HG for 48 h. **A.** Protein expression levels of CDK2 in NRCMs after transfection with CDK2 siRNA (n=6, *P<0.05 *vs.* the ScRNA group). **B**. HMGA1 expression levels in NRCMs (n=6). **C.** mRNA levels of pro-inflammatory markers in NRCMs (n=6). **D.** TUNEL staining and quantification results in NRCMs (n=5). *P<0.05 *vs.* the CON group; #P<0.05 *vs.* the HG group.

**E-H.** NRCMs were transfected with Ad-P27 and Ad-HMGA1 and then stimulated with HG for 48 h. **E.** Protein expression levels of CP27 in NRCMs after transfection with Ad-P27 (n=6, *P<0.05 *vs.* the Ad-NC group). **F**. HMGA1 expression levels in NRCMs (n=6). **G.** mRNA levels of pro-inflammatory markers in NRCMs (n=6). **H.** TUNEL staining and quantification results in NRCMs (n=5). *P<0.05 *vs.* the CON group; #P<0.05 *vs.* the HG group. All the in vitro experiments were performed independently for 3 times.
