# Supplementary material for: High-mobility group AT-hook 1 promotes cardiac dysfunction in diabetic cardiomyopathy via autophagy inhibition
Source: Cell Death Dis. 2020 Mar 2;11(3):160. doi: 10.1038/s41419-020-2316-4 (PMC7052237; doi:10.1038/s41419-020-2316-4)
Supplement: Supplementary file 4 — Supplementary Material-2 [file 41419_2020_2316_MOESM4_ESM.docx]

**Supplementary materials**

**Table**

Table S1: Primer sequences used for RT-PCR

| mRNA | Forward | Reverse |
| --- | --- | --- |
| IL-1^a^ | CCGTGGACCTTCCAGGATGA | GGGAACGTCACACACCAGCA |
| IL-6^a^ | AGTTGCCTTCTTGGGACTGA | TCCACGATTTCCCAGAGAAC |
| TNFa^a^ | ACTGAACTTCGGGGTGATCGGT | TGGTTTGCTACGACGTGGGCTA |
| GAPDH^a^ | ACTCCACTCACGGCAAATTC | TCTCCATGGTGGTGAAGACA |
| IL-1^b^ | GGGATGATGACGACCTGCTAG | ACCACTTGTTGGCTTATGTTCTG |
| IL-6^b^ | GTTGCCTTCTTGGGACTGATG | ATACTGGTCTGTTGTGGGTGGT |
| TNFα^b^ | AGCATGATCCGAGATGTGGAA | TAGACAGAAGAGCGTGGTGGC |
| GAPDH ^b^ | GACATGCCGCCTGGAGAAAC | AGCCCAGGATGCCCTTTAGT |

Sequences are listed 5'–3'.
^a^ The PCR used the primers in mice.

^b^ The PCR used the primers in Neonatal rat cardiomyocytes.

Table S2: The information of the primary antibodies used in western blot

| Antibody description | Source | No. and dilution |
| --- | --- | --- |
| HMGA1 | Abcam | # ab129153, 1:1000 |
| Bax | Cell Signaling Technology | #2722, 1:1000 |
| Bcl-2 | Cell Signaling Technology | # 2870, 1:1000 |
| Cytochrome C | Cell Signaling Technology | # 4272, 1:1000 |
| Atg7 | Cell Signaling Technology | #2613, 1:1000 |
| P62 | Abcam | #ab91526, 1:1000 |
| LC3 | Cell Signaling Technology | #12741, 1:1000 |
| P-AMPKa | Cell Signaling Technology | # 2535, 1:1000 |
| T-AMPKa | Cell Signaling Technology | # 2603P, 1:1000 |
| P-AKT | Cell Signaling Technology | # 4060, 1:1000 |
| T-AKT | Cell Signaling Technology | # 4691, 1:1000 |
| P-ERK1/2 | Cell Signaling Technology | 4370P, 1:1000 |
| T-ERK1/2 | Cell Signaling Technology | 4695, 1:1000 |
| P-mTOR | Cell Signaling Technology | 2971, 1:1000 |
| T-mTOR  P-4EBP1  T-4EBP1 | Cell Signaling Technology  Cell Signaling Technology  Cell Signaling Technology | 2983, 1:1000  2955, 1:1000  9644, 1:1000 |
| P27 | Cell Signaling Technology | 2552, 1:1000 |
| CDK2 | Cell Signaling Technology | 2546P, 1:1000 |
| GAPDH | Cell Signaling Technology | #2118, 1:1000 |

**Table S3.** Echocardiography and hemodynamics Parameters in diabetic mice after injected with AAV9-HMGA1

|  | AAV9-NC  CON  (n=8-10) | AAV9-HMGA1  CON  (n=8-10) | AAV9-NC  DCM  (n=10) | AAV9-HMGA1 DCM  (n=10) |
| --- | --- | --- | --- | --- |
| LVEF (%) | 68.0±6.1 | 66. 4±5.6 | 53.1±7.3* | 42.0±8.9*# |
| LVFS (%) | 31.8±4.3 | 30.7±3.8 | 22.5±3.8* | 16.8±4.2*# |
| HR (bpm) | 452±21 | 446±27 | 453±39 | 441±58 |
| dp/dt max (mmHg/s) | 9491±761 | 9496±789 | 6905±750* | 4501±577*# |
| dp/dt min(mmHg/s)  Tau (Weiss; ms) | -9056±849  8.34±1.29 | -9065±693  9.24±1.06 | -6818±968*  13.94±1.44* | -4330±682*#  18.83±1.59*# |

LVEF, left ventricular ejection fraction; LVFS, left ventricular ejection of shortening; HR, heart rate; dp/dtmax, maximal rate of pressure development; dp/dtmin, maximal rate of pressure decay; Tau, time constant of LV pressure decay *P<0.05 for difference from corresponding CON group. #P<0.05 vs AVV9-NC-DCM group.

**Table S4.** Echocardiography and hemodynamics Parameters in diabetic mice after injected with AAV9-shHMGA1

|  | AAV9-ScRNA  CON  (n=8-10) | AAV9-shHMGA1  CON  (n=8-10) | AAV9-ScRNA  DCM  (n=10) | AAV9-shHMGA1 DCM  (n=10) |
| --- | --- | --- | --- | --- |
| LVEF (%) | 68.9±4.7 | 67.7±5.0 | 45.8±3.2* | 55.2±4.6*# |
| LVFS (%) | 31.7±3.4 | 31.5±3.6 | 18.5±1.6* | 23.6±2.6*# |
| HR (bpm) | 455±21 | 445±29 | 459±26 | 457±22 |
| dp/dt max (mmHg/s) | 9480±481 | 9581±561 | 6331±438* | 7803±522*# |
| dp/dt min(mmHg/s)  Tau (Weiss; ms) | -9762±793  8.27±0.77 | -9523±897  8.42±1.04 | -6284±611*  14.93±1.72* | -7824±775*#  10.95±0.86*# |

LVEF, left ventricular ejection fraction; LVFS, left ventricular ejection of shortening; HR, heart rate; dp/dtmax, maximal rate of pressure development; dp/dtmin, maximal rate of pressure decay; Tau, time constant of LV pressure decay *P<0.05 for difference from corresponding CON group. #P<0.05 vs AAV9-ScRNA-DCM group.

**Table S5.** Echocardiography and hemodynamics Parameters in diabetic mice after injected with AAV9-HMGA1 and AAV9-P27

|  | AAV9-NC  AVV9-P27  CON  (n=8) | AAV9-HMGA1  AVV9-P27  CON  (n=8) | AAV9-NC  AVV9-P27  DCM  (n=8) | AAV9-HMGA1 AVV9-P27  DCM  (n=8) |
| --- | --- | --- | --- | --- |
| LVEF (%) | 65.6±4.5 | 65.4±5.3 | 56.1±4.3* | 57.5±6.8* |
| LVFS (%) | 30.1±3.0 | 29.9±3.6 | 24.1±2.5* | 24.9±4.0* |
| HR (bpm) | 458±22 | 464±53 | 459±79 | 478±51 |
| dp/dt max (mmHg/s) | 9410±458 | 9648±444 | 5866±343* | 5832±458* |
| dp/dt min(mmHg/s)  Tau (Weiss; ms) | -9640±796  8.04±1.19 | -9539±896  8.18±1.06 | -5830±496*  12.9±2.27* | -5823±361*  12.7±1.44* |

LVEF, left ventricular ejection fraction; LVFS, left ventricular ejection of shortening; HR, heart rate; dp/dtmax, maximal rate of pressure development; dp/dtmin, maximal rate of pressure decay; Tau, time constant of LV pressure decay *P<0.05 for difference from corresponding CON group. #P<0.05 vs AVV9-NC-DCM group.
